# 2-Acetyl­hydrazono-2-phenyl­aceto­hydrazide

**DOI:** 10.1107/S160053680802624X

**Published:** 2008-08-23

**Authors:** Bai-Cheng Feng, Zhi Yang, Xu Yi

**Affiliations:** a College of Chemical Engineering, Qingdao University of Science and Technology, Qingdao 266042, People’s Republic of China; b College of Chemistry and Molecular Engineering, Qingdao University of Science and Technology, Qingdao 266042, People’s Republic of China

## Abstract

The title compound, C_10_H_12_N_4_O_2_, was prepared as an inter­mediate for the synthesis of metamitron. The benzene ring plane forms dihedral angles of 66.0 (1) and 3.5 (5)° with the hydrazine plane and the acetyl­imino plane, respectively. The crystal structure involves inter­molecular N—H⋯O hydrogen bonds.

## Related literature

For related literature on the biological activity, see: Javier *et al.* (2006 ▶). For a similar structure, see: Glaser *et al.* (1993 ▶). For the preparation, see: Pan *et al.* (2007 ▶).
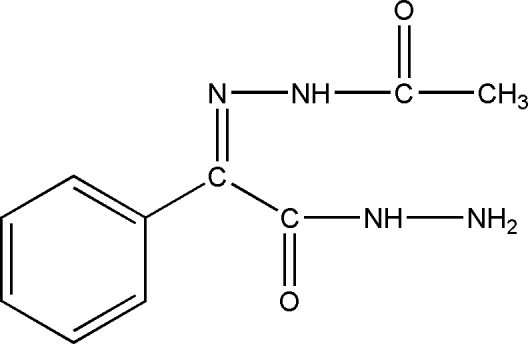


## Experimental

### 

#### Crystal data

C_10_H_12_N_4_O_2_
*M* *_r_* = 220.24
Monoclinic, 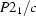

*a* = 12.737 (3) Å
*b* = 4.5867 (10) Å
*c* = 21.002 (7) Å
β = 117.62 (2)°
*V* = 1087.1 (5) Å^3^
*Z* = 4
Mo *K*α radiation
μ = 0.10 mm^−1^
*T* = 153 (2) K
0.42 × 0.31 × 0.22 mm

#### Data collection

Rigaku R-AXIS RAPID IP area-detector diffractometer
Absorption correction: multi-scan (*ABSCOR*; Higashi 1995 ▶) *T*_min_ = 0.804, *T*_max_ = 0.979
7793 measured reflections
1878 independent reflections
1624 reflections with *I* > 2σ(*I*)
*R*_int_ = 0.024

#### Refinement

*R*[*F* ^2^ > 2σ(*F* ^2^)] = 0.035
*wR*(*F* ^2^) = 0.103
*S* = 1.08
1878 reflections
155 parameters
H atoms treated by a mixture of independent and constrained refinement
Δρ_max_ = 0.20 e Å^−3^
Δρ_min_ = −0.16 e Å^−3^

### 

Data collection: *RAPID-AUTO* (Rigaku, 2004 ▶); cell refinement: *RAPID-AUTO*; data reduction: *RAPID-AUTO*; program(s) used to solve structure: *SHELXTL* (Sheldrick, 2008 ▶); program(s) used to refine structure: *SHELXTL*; molecular graphics: *SHELXTL*; software used to prepare material for publication: *SHELXTL*.

## Supplementary Material

Crystal structure: contains datablocks I, global. DOI: 10.1107/S160053680802624X/bv2104sup1.cif

Structure factors: contains datablocks I. DOI: 10.1107/S160053680802624X/bv2104Isup2.hkl

Additional supplementary materials:  crystallographic information; 3D view; checkCIF report

## Figures and Tables

**Table 1:** Hydrogen-bond geometry (Å, °)

*D*—H⋯*A*	*D*—H	H⋯*A*	*D*⋯*A*	*D*—H⋯*A*
N2—H2*B*⋯O2^i^	0.88	2.09	2.9512 (16)	167
N3—H3*B*⋯O1^ii^	0.88	2.08	2.8450 (15)	145
N4—H4*B*⋯O2^i^	0.90 (2)	2.396 (19)	3.0903 (19)	134.5 (15)
N4—H4*C*⋯O1^iii^	0.890 (18)	2.274 (18)	3.0514 (19)	145.8 (15)

Symmetry codes: (i) 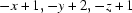; (ii) 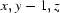; (iii) 
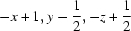
.

**Fig. 1.**
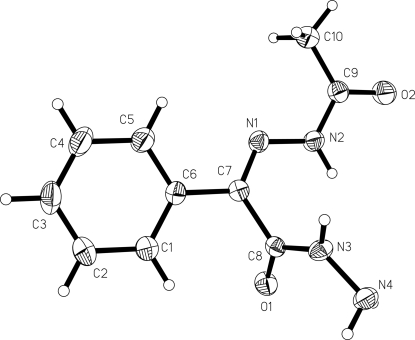
View of the title compound (I), with displacement ellipsoids drawn at the 35% probability level.

**Fig. 2.**
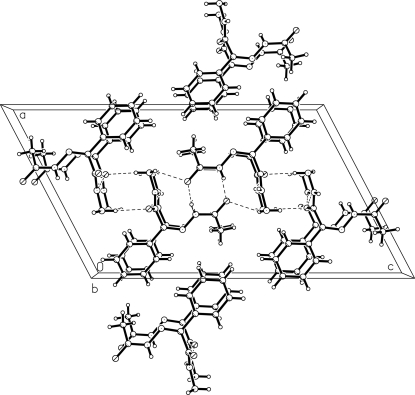
A packing diagram of the molecule of the title compound, view down b axis. Hydrogen bonds are shown as dashed lines.

## supplementary crystallographic information

### Comment

Metamitron (Trade name: Goltix) is used against grass and broad-leaved weeds in sugar and fodder beets. Metamitron is applied pre-drilling and pre- and post-emergence (post-emergence as sequential treatment tank-mixed with oil or other herbicides). Metamitron is also used in mangold, red beet and certain strawberry varieties. The dose rates for metamitron are 0.35–4.2 kg active ingredient/ha for all crops.The currently used weed control strategy in sugarbeet involves a mixture of herbicides (phenmedipham, ethofumesate, metamitron, chloridazon *etc*) to control dicotyledonus weeds. Wettable powder (70%) has been used for the control of morel goosefoot chickweed Lamium barbatum *etc*. Metamitron can be used before and after planting. It can be applied to the control of the entire crop growing period with better efficacy when it cooperates with others herbicides and pesticides (Javier *et al.*, 2006).

The title compound (I) was synthesized as an intermediate for the synthesis of metamitron. We report here the crystal structure of (I).

In (I) (Fig. 1), all bond lengths and angles are normal and in a good agreement with those reported previously (Glaser *et al.*, 1993). The benzene ring plane forms dihedral angles of 66.0 (1)° and 3.5 (5)° with the hydrazine plane consisting of O1, N3, N4, and C8, and the acetylimino plane consisting of O2, N1, N2, C9, and C10, respectively. The crystal structure is stabilized by intermolecular N–H–O hydrogen bonds.

### Experimental

Phenylglyoxylic acid ethyl ester 2-acetylhydrazone 23.4 g (0.1 mol), was dissolved in 100 ml ethanol in a flask equipped with stirrer and reflux condenser. Hydrazine hydrate 7.5 g (0.1 mmol) was slowly added from a dropping-funnel during 30 minutes while maintaining the temperature at 25–30°C for two hours. Portions of the solvent were distilled and the remaining solution cooled in ice water. White crystals separated out after a short time (18.9 g, yield 87.3%) (Pan *et al.*, 2007). Single crystals suitable for X-ray measurement were obtained by recrystallization from petrol ether at room temperature.

### Refinement

All H atoms were found on difference maps. The hydrazine H atoms were refined freely, giving an N—H bond distance of 0.89 or 0.90 Å. The remaining H atoms were positioned geometrically [N—H = 0.88 Å C—H = 0.95 Å (CH), C—H = 0.98 Å (CH3), and*U*_iso_ (H) = 1.5 times (Methyl) or *U*_iso_(H) = 1.2 times (other H atoms)].

### Crystal data

**Table:**

C_10_H_12_N_4_O_2_	*F*_000_ = 464
*M**_r_* = 220.24	*D*_x_ = 1.346 Mg m^−^^3^
Monoclinic, *P*2_1_/*c*	Mo *K*α radiation λ = 0.71073 Å
Hall symbol: -P 2ybc	Cell parameters from 2652 reflections
*a* = 12.737 (3) Å	θ = 2.6–25.6º
*b* = 4.5867 (10) Å	µ = 0.10 mm^−^^1^
*c* = 21.002 (7) Å	*T* = 153 (2) K
β = 117.62 (2)º	Block, colorless
*V* = 1087.1 (5) Å^3^	0.42 × 0.31 × 0.22 mm
*Z* = 4	

### Data collection

**Table:**

Rigaku R-AXIS RAPID IP area-detector diffractometer	1878 independent reflections
Radiation source: Rotating Anode	1624 reflections with *I* > 2σ(*I*)
Monochromator: graphite	*R*_int_ = 0.024
*T* = 153(2) K	θ_max_ = 25.0º
ω Oscillation scans	θ_min_ = 3.2º
Absorption correction: multi-scan(ABSCOR; Higashi 1995)	*h* = −15→15
*T*_min_ = 0.805, *T*_max_ = 0.979	*k* = −5→5
7793 measured reflections	*l* = −24→24

### Refinement

**Table:**

Refinement on *F*^2^	Hydrogen site location: inferred from neighbouring sites
Least-squares matrix: full	H atoms treated by a mixture of independent and constrained refinement
*R*[*F*^2^ > 2σ(*F*^2^)] = 0.035	*w* = 1/[σ^2^(*F*_o_^2^) + (0.0567*P*)^2^ + 0.2207*P*] where *P* = (*F*_o_^2^ + 2*F*_c_^2^)/3
*wR*(*F*^2^) = 0.103	(Δ/σ)_max_ = 0.001
*S* = 1.08	Δρ_max_ = 0.20 e Å^−^^3^
1878 reflections	Δρ_min_ = −0.16 e Å^−^^3^
155 parameters	Extinction correction: SHELXTL (Sheldrick, 2008), Fc^*^=kFc[1+0.001xFc^2^λ^3^/sin(2θ)]^-1/4^
Primary atom site location: structure-invariant direct methods	Extinction coefficient: 0.024 (4)
Secondary atom site location: difference Fourier map	

### Special details

**Table:**

Geometry. All e.s.d.'s (except the e.s.d. in the dihedral angle between two l.s. planes) are estimated using the full covariance matrix. The cell e.s.d.'s are taken into account individually in the estimation of e.s.d.'s in distances, angles and torsion angles; correlations between e.s.d.'s in cell parameters are only used when they are defined by crystal symmetry. An approximate (isotropic) treatment of cell e.s.d.'s is used for estimating e.s.d.'s involving l.s. planes.
Refinement. Refinement of *F*^2^ against ALL reflections. The weighted *R*-factor *wR* and goodness of fit *S* are based on *F*^2^, conventional *R*-factors *R* are based on *F*, with *F* set to zero for negative *F*^2^. The threshold expression of *F*^2^ > σ(*F*^2^) is used only for calculating *R*-factors(gt) *etc*. and is not relevant to the choice of reflections for refinement. *R*-factors based on *F*^2^ are statistically about twice as large as those based on *F*, and *R*- factors based on ALL data will be even larger.

### Fractional atomic coordinates and isotropic or equivalent isotropic displacement parameters (Å^2^)

**Table:**

	*x*	*y*	*z*	*U*_iso_*/*U*_eq_	
O1	0.40065 (9)	1.19619 (19)	0.29348 (5)	0.0427 (3)	
O2	0.44401 (10)	0.7702 (2)	0.54459 (5)	0.0531 (3)	
N1	0.27633 (9)	0.7290 (2)	0.35864 (6)	0.0362 (3)	
N2	0.36620 (10)	0.8004 (3)	0.42552 (6)	0.0390 (3)	
H2B	0.4245	0.9138	0.4292	0.047*	
N3	0.49466 (9)	0.7681 (2)	0.33409 (6)	0.0359 (3)	
H3B	0.4858	0.5829	0.3415	0.043*	
N4	0.60867 (10)	0.8674 (3)	0.34944 (7)	0.0423 (3)	
C1	0.19350 (13)	0.8340 (4)	0.17011 (7)	0.0469 (4)	
H1A	0.2573	0.9530	0.1742	0.056*	
C2	0.10157 (15)	0.7697 (4)	0.10282 (8)	0.0573 (4)	
H2A	0.1027	0.8455	0.0610	0.069*	
C3	0.00929 (13)	0.5978 (4)	0.09616 (8)	0.0585 (5)	
H3A	−0.0536	0.5542	0.0499	0.070*	
C4	0.00809 (14)	0.4882 (4)	0.15689 (9)	0.0634 (5)	
H4A	−0.0560	0.3691	0.1524	0.076*	
C5	0.09888 (13)	0.5496 (4)	0.22391 (8)	0.0517 (4)	
H5A	0.0975	0.4711	0.2654	0.062*	
C6	0.19282 (11)	0.7257 (3)	0.23147 (7)	0.0348 (3)	
C7	0.28992 (11)	0.7937 (3)	0.30347 (6)	0.0321 (3)	
C8	0.40034 (10)	0.9387 (3)	0.30915 (6)	0.0301 (3)	
C9	0.36513 (12)	0.6973 (3)	0.48520 (7)	0.0378 (3)	
C10	0.26833 (13)	0.4954 (4)	0.47702 (8)	0.0514 (4)	
H10A	0.2836	0.4216	0.5243	0.077*	
H10B	0.2650	0.3317	0.4462	0.077*	
H10C	0.1926	0.5998	0.4552	0.077*	
H4C	0.6083 (14)	0.900 (4)	0.3076 (10)	0.055 (5)*	
H4B	0.6174 (15)	1.039 (4)	0.3717 (9)	0.061 (5)*	

### Atomic displacement parameters (Å^2^)

**Table:**

	*U*^11^	*U*^22^	*U*^33^	*U*^12^	*U*^13^	*U*^23^
O1	0.0494 (6)	0.0282 (5)	0.0425 (5)	−0.0037 (4)	0.0144 (4)	0.0028 (4)
O2	0.0597 (7)	0.0632 (7)	0.0316 (5)	−0.0165 (5)	0.0171 (5)	−0.0079 (5)
N1	0.0332 (6)	0.0421 (7)	0.0320 (6)	−0.0033 (4)	0.0138 (5)	−0.0042 (5)
N2	0.0379 (6)	0.0480 (7)	0.0313 (6)	−0.0107 (5)	0.0162 (5)	−0.0060 (5)
N3	0.0363 (6)	0.0275 (5)	0.0472 (6)	−0.0034 (4)	0.0220 (5)	0.0000 (5)
N4	0.0367 (6)	0.0480 (8)	0.0463 (7)	−0.0055 (5)	0.0227 (5)	−0.0012 (6)
C1	0.0449 (8)	0.0560 (9)	0.0371 (7)	−0.0082 (7)	0.0166 (6)	−0.0022 (7)
C2	0.0578 (10)	0.0741 (12)	0.0323 (7)	−0.0047 (8)	0.0144 (7)	−0.0015 (7)
C3	0.0421 (9)	0.0794 (12)	0.0399 (8)	−0.0050 (8)	0.0070 (6)	−0.0153 (8)
C4	0.0437 (9)	0.0879 (13)	0.0532 (9)	−0.0243 (8)	0.0180 (7)	−0.0173 (9)
C5	0.0452 (8)	0.0679 (11)	0.0417 (7)	−0.0158 (7)	0.0200 (6)	−0.0075 (7)
C6	0.0316 (7)	0.0370 (7)	0.0339 (7)	0.0014 (5)	0.0136 (5)	−0.0044 (5)
C7	0.0343 (7)	0.0295 (7)	0.0323 (6)	0.0009 (5)	0.0155 (5)	−0.0023 (5)
C8	0.0364 (7)	0.0274 (7)	0.0249 (6)	−0.0032 (5)	0.0128 (5)	−0.0041 (5)
C9	0.0406 (7)	0.0401 (8)	0.0331 (7)	0.0008 (6)	0.0176 (6)	−0.0023 (6)
C10	0.0490 (9)	0.0602 (10)	0.0434 (8)	−0.0078 (7)	0.0200 (7)	0.0072 (7)

### Geometric parameters (Å, °)

**Table:**

O1—C8	1.2263 (15)	C2—C3	1.368 (2)
O2—C9	1.2308 (17)	C2—H2A	0.9500
N1—C7	1.2833 (17)	C3—C4	1.378 (2)
N1—N2	1.3783 (16)	C3—H3A	0.9500
N2—C9	1.3455 (18)	C4—C5	1.374 (2)
N2—H2B	0.8800	C4—H4A	0.9500
N3—C8	1.3218 (16)	C5—C6	1.391 (2)
N3—N4	1.4093 (15)	C5—H5A	0.9500
N3—H3B	0.8800	C6—C7	1.4762 (18)
N4—H4C	0.890 (18)	C7—C8	1.5097 (17)
N4—H4B	0.90 (2)	C9—C10	1.487 (2)
C1—C6	1.385 (2)	C10—H10A	0.9800
C1—C2	1.386 (2)	C10—H10B	0.9800
C1—H1A	0.9500	C10—H10C	0.9800
			
C7—N1—N2	117.99 (11)	C4—C5—C6	120.46 (15)
C9—N2—N1	120.24 (11)	C4—C5—H5A	119.8
C9—N2—H2B	119.9	C6—C5—H5A	119.8
N1—N2—H2B	119.9	C1—C6—C5	118.57 (12)
C8—N3—N4	123.36 (11)	C1—C6—C7	120.92 (12)
C8—N3—H3B	118.3	C5—C6—C7	120.51 (12)
N4—N3—H3B	118.3	N1—C7—C6	118.45 (12)
N3—N4—H4C	107.2 (11)	N1—C7—C8	122.77 (11)
N3—N4—H4B	105.6 (11)	C6—C7—C8	118.78 (11)
H4C—N4—H4B	108.0 (16)	O1—C8—N3	124.14 (12)
C6—C1—C2	120.39 (14)	O1—C8—C7	121.45 (11)
C6—C1—H1A	119.8	N3—C8—C7	114.37 (10)
C2—C1—H1A	119.8	O2—C9—N2	119.50 (13)
C3—C2—C1	120.44 (15)	O2—C9—C10	122.00 (12)
C3—C2—H2A	119.8	N2—C9—C10	118.49 (12)
C1—C2—H2A	119.8	C9—C10—H10A	109.5
C2—C3—C4	119.58 (14)	C9—C10—H10B	109.5
C2—C3—H3A	120.2	H10A—C10—H10B	109.5
C4—C3—H3A	120.2	C9—C10—H10C	109.5
C5—C4—C3	120.55 (15)	H10A—C10—H10C	109.5
C5—C4—H4A	119.7	H10B—C10—H10C	109.5
C3—C4—H4A	119.7		
			
C7—N1—N2—C9	−169.43 (11)	C5—C6—C7—N1	−11.5 (2)
C6—C1—C2—C3	−0.2 (3)	C1—C6—C7—C8	−10.55 (19)
C1—C2—C3—C4	0.0 (3)	C5—C6—C7—C8	169.07 (13)
C2—C3—C4—C5	−0.2 (3)	N4—N3—C8—O1	3.72 (19)
C3—C4—C5—C6	0.6 (3)	N4—N3—C8—C7	−174.25 (11)
C2—C1—C6—C5	0.7 (2)	N1—C7—C8—O1	−106.29 (15)
C2—C1—C6—C7	−179.71 (14)	C6—C7—C8—O1	73.11 (15)
C4—C5—C6—C1	−0.9 (2)	N1—C7—C8—N3	71.75 (15)
C4—C5—C6—C7	179.52 (15)	C6—C7—C8—N3	−108.85 (13)
N2—N1—C7—C6	−178.31 (11)	N1—N2—C9—O2	−177.76 (12)
N2—N1—C7—C8	1.09 (19)	N1—N2—C9—C10	2.8 (2)
C1—C6—C7—N1	168.88 (13)		

### Hydrogen-bond geometry (Å, °)

**Table:**

*D*—H···*A*	*D*—H	H···*A*	*D*···*A*	*D*—H···*A*
N2—H2B···O2^i^	0.88	2.09	2.9512 (16)	167
N3—H3B···O1^ii^	0.88	2.08	2.8450 (15)	145
N4—H4B···O2^i^	0.90 (2)	2.396 (19)	3.0903 (19)	134.5 (15)
N4—H4C···O1^iii^	0.890 (18)	2.274 (18)	3.0514 (19)	145.8 (15)

Symmetry codes: (i) −*x*+1, −*y*+2, −*z*+1; (ii) *x*, *y*−1, *z*; (iii) −*x*+1, *y*−1/2, −*z*+1/2.
